# Advancing Public Health through Community Pharmacy Practice

**DOI:** 10.3390/pharmacy11020056

**Published:** 2023-03-15

**Authors:** Natalie DiPietro Mager, David Bright

**Affiliations:** 1 Department of Pharmacy Practice, Raabe College of Pharmacy, Ohio Northern University, Ada, OH 45810, USA; 2 Department of Pharmaceutical Sciences, Ferris State University College of Pharmacy, Big Rapids, MI 49307, USA

The overarching goal of public health is to advance the health of individuals, communities, and populations [1]. This is accomplished through various methods such as research, policy, education, and prevention strategies usually characterized as primary (i.e., preventing the development of disease), secondary (i.e., the early detection of disease), or tertiary (i.e., slowing the progression of disease) [1,2,3]. Public health initiatives have improved not only the length but also the quality of life for community members and have often resulted in cost savings to healthcare systems and societies [1].

Pharmacists are highly skilled medication experts who can directly affect public health. Prominent organizations worldwide have supported roles for pharmacists in improving and protecting public health [4,5,6,7] as the profession continues to build upon and refine necessary competencies [8,9]. Due to the nature of their work, pharmacists impact both patient- and population-level health outcomes, spanning the “micro” to “macro” levels of influence in public health [5,6,10,11,12]. Examples of the important roles pharmacists have played in public health initiatives in the United States (U.S.) include contributing to the Million Hearts Campaign [13], facilitating the National Diabetes Prevention Program [14,15], and working to meet Healthy People objectives [16], among countless others. Furthermore, as members of interprofessional teams, pharmacists’ provision of clinical services has improved patient and population health outcomes [17]. Pharmacists have also become increasingly involved in screening patients for social determinants of health and performing required interventions or referrals as part of clinical–community linkages [18,19,20,21,22,23].

Whereas this impact can be seen across a variety of health care settings and systems, pharmacists practicing in community pharmacies have the unique potential to make public health interventions. Often known traditionally as “drug stores,” community pharmacies create opportunities for patients to quickly access the health care system, often because of their community-based locations that reduce potential barriers due to travel distance or a lack of transportation [24]. Not only do many community pharmacies offer opportunities for direct, brief interactions between a patient and a pharmacist without an appointment or fee, but also often provide other non-dispensing service interventions such as immunizations (generally associated with sustainable fees or reimbursement). In this way, the strong accessibility of community pharmacists is leveraged to drive high volumes of interventions [25].

The engagement of community pharmacists in non-dispensing services has been quite impressive in recent years [26]. For instance, the COVID-19 pandemic has increased attention to pharmacists’ contributions to public health, and many community pharmacists advanced services and programs during this time [27,28,29,30,31,32]. Additionally, over 300 million COVID vaccine doses were administered through community pharmacies in the U.S. by February 2023 [33]. Non-dispensing services create key opportunities for community pharmacists at large to make significant public health impacts. However, it can be challenging for a single pharmacist in a single pharmacy to perceive that impact and prioritize non-dispensing services when the medication dispensing process can be substantially time-consuming and fraught with tight reimbursement margins.

This Special Issue serves to provide some of the latest advancements in community pharmacists’ support of public health activities. The range of topics of the papers in this Special Issue shows the broad reach of community pharmacists into the many potential facets of public health. However, although diversity among the papers exists, there are also several common themes that the papers exemplify.

The first common theme that emerged is that when developing pharmacist-provided services that advance public health, we must be sure to engage not only the pharmacy workforce but also pharmacy patients [34,35,36]. Previously published literature has indicated low awareness or interest among patients regarding some non-dispensing services available at community pharmacies in the U.S., for example [37]. Therefore, we must make sure that we develop services that will work for them and meet their needs [34,35,36]. Likewise, we must promote these services well to community members to ensure uptake and ultimately improve public health [34]. 

The second theme that emerged revolved around the rapidly increasing variety of methods that community pharmacists are using to enhance access to health care services. For instance, over-the-counter hearing aids are a very recent addition to the repertoire of community pharmacies in the U.S. and have offered the ability to reduce barriers that prevent patients from mitigating hearing deficits [38]. The shifting regulatory environment has further empowered community pharmacists to engage in contraceptive and maternal health service provision in the U.S. [39], with other countries also looking to determine how best to provide such services [40]. Finally, COVID-related services, including travel medicine support, have seen a rapid increase in community pharmacies since the beginning of the pandemic [36,41]. Each of these topics is specifically illustrated in this Special Issue and represents examples of logical extensions of the community pharmacist’s existing abilities to make public health interventions. 

As valuable as service implementation can be, without addressing the logistical and workflow realities, implementation can be substantially difficult. The third theme that emerged among the papers in this Special Issue involved strategies to address clinical and logistical efficiency so that services could be provided on a practical basis. Strategies such as more thoroughly weaving electronic health records (EHR) into the pharmacy’s existing processes [42] or incorporating a health risk assessment into the pharmacy’s workflow [43] illustrate the intersection of practical efficiencies with the advancement of service provision. As limited time and funding can often be a barrier for community pharmacists to make public health interventions, we hope that addressing these issues can not only help service implementation but also service sustainability. 

Of note, many of the papers featured in this Special Issue included trainee authors. We appreciate that experienced practitioners and researchers are investing in the next generation of public health pharmacy advocates by engaging them in the research process. The breadth of trainee level was also encouraging, with examples including high school students [41], pharmacy students [35], post-doctoral residents [35,38,43], and graduate students [35]. This is positive for the advancement of public health services and, because many of these trainees are or will become pharmacists, the integration of pharmacy practice and public health can be further strengthened [44]. Moreover, as some regions of the world such as the U.S. are currently seeing a decline in prospective students interested in pursuing pharmacy education, involving high school students in meaningful pharmacy-related projects may be a way to attract students to the profession of pharmacy and serve as a pipeline to recruit talented and dedicated students. Similarly, engaging pharmacy students in interesting and timely public health-related projects may help with retention and expose them to areas of the profession that they may want to pursue after graduation. 

Lastly, the need to disseminate information regarding pharmacists’ impact on public health to audiences outside of the pharmacy community itself has been recognized [12]. This is important as it shares best practices and lessons learned as well as brings visibility to other stakeholders regarding possible partnerships with pharmacists. The open access format of this journal facilitates the exchange of ideas across boundaries, whether they are by discipline, geography, or culture. This serves to raise awareness among multiple audiences about the contributions of community pharmacy to public health and opportunities for collaboration.

Community pharmacists have long been recognized as integral to advancing public health. We hope that this new combination of papers that address a variety of topics at the intersection of community pharmacy and public health provides value towards the implementation and improvement of constructive interventions. Although it may at times feel intimidating to develop or expand a pharmacy service, we hope that the methods shared in this Special Issue, along with other published guides [45,46,47], will be helpful resources. Although the majority of works in this issue come from the U.S., international works are also included, demonstrating that community pharmacists can make a positive impact on public health regardless of the diverse roles, regulations, and customs across pharmacy practice in different countries. We are optimistic that readers can extrapolate the pockets of excellence described throughout this Special Issue to apply to how pharmacy is practiced in their hometown, and that ideas for innovative pharmacy practice interventions can be stimulated for implementation by clinical and community leaders.
